# Significance and current approaches to vascular graft infection

**DOI:** 10.1007/s12055-023-01638-w

**Published:** 2023-12-04

**Authors:** Carlos–Alberto Mestres, Mathias Van Hemelrijck, Eduard Quintana, Francis Edwin Smit

**Affiliations:** 1 Department of Cardiothoracic Surgery, Faculty of Health Sciences and The Robert WM Frater Cardiovascular Research Centre, The University of the Free State, PO Box 339 (Internal Box G32), Bloemfontein, 9300 South Africa; 2 Department of Cardiac Surgery, University Hospital Zurich, Zurich, Switzerland; 3 Department of Cardiovascular Surgery, Hospital Clinic, University of Barcelona, Barcelona, Spain

**Keywords:** Vascular infection, Prosthetic graft, Cardiovascular surgery, Infectious diseases, Antimicrobial therapy

## Abstract

Vascular graft/endograft infection (VGEI) is a constant in cardiovascular surgery with published rates between 1 and 5%. Every graft type and anatomical location is a potential target for infectious complications. These patients are sick patients with high frailty burden. Management of VGEI entails a multidisciplinary and multimodality approach. Here we review some aspects of the problem of VGEI including prevention, diagnosis, and surgical therapy with focus on recent developments in the field.

## Introduction

It is a known fact that any implant placed in the human body is susceptible to presenting complications immediately after the intervention, of whatever type, or during the follow-up of the patient, that is, during his/her lifespan. This is an old story and may eventually develop at any patient age and, as said, after any type of implant [1, 2]. Antibiotic prophylaxis before any implant is currently accepted as routine surgical practice. The goal of administering antibiotics before an operation is having the highest concentration in tissues at the time of the initiation and during surgery [3, 4] and thus reducing the risk of infections that may develop postoperatively.

Infection is a bad traveling companion for the surgeon regardless of the specialty. This is because of the high morbidity and mortality related to any infection starting with surgical site infection [5, 6]. There is mounting evidence supporting the routine preoperative antibiotic prophylaxis in surgery and especially when an implant is contemplated [7]. This, of course, also applies to cardiovascular surgery [8, 9]. In this contribution, we will address the significance and current approaches to vascular graft and endograft infection (VGEI) due to the potential catastrophic consequences of these aggressive infections.

## Methods

This is a narrative review in which concepts and articles related to VGEI that should be of interest for practising cardiovascular surgeons and allied professionals with experience and expertise are considered. Topics include current knowledge, prevention and diagnosis, and replacement materials used in vascular practice. Search terms included “vascular graft”, “infection”, “surgery”, and “antimicrobial therapy”. Historical and contemporary articles have been selected to understand developments in the field. For practical purposes, focus has been placed on intrathoracic prostheses.

## The knowledge

VGEIs are uncommon, at an estimated 1–3% reported rate after open surgical implantation. Mortality is high, in the range of 20–30% [10, 11]. Although infection rates may change according to series, VGEI of any location is a potentially lethal entity. In addition to this high toll, it is associated with relevant health costs [12, 13].

### The questions

There are two main questions when it comes to VGEI. *The first question*: when does the infection occur? There is evidence that VGEI starts preoperatively by inoculation with bacteria from the patient’s skin flora at the time of surgery. This has been well addressed by Hasse et al. [10] and Van Hemelrijck et al. [14]. This is particularly significant in the case of prosthetic vascular graft infection (VGI) in the groin due to surgical site infection [10] and continues to be a major issue in vascular surgery.

Well-known risk factors for VGEIs include groin incisions, wound infections, and comorbidities typical of patients with vascular disease. Modifiable predictors for VGEIs as targets for infection prevention strategies have been well addressed by Anagnostopoulos et al. in a recent seminal contribution [15]. In their prospective Swiss Vascular Graft Infection Cohort (VASGRA), they analyzed 438 predominantly male (83.1%) patients with a median age of 71 years totalling 554 person/years of follow-up. The authors identified incisional surgical site infections, hemorrhage, renal insufficiency, inadequate perioperative prophylaxis, and procedural time increases of 1-h intervals to be risk factors for VGEIs. These data confirm that several postsurgical infectious and non-infectious complications are modifiable predictive factors for VGEIs. This is relevant information [15].

The *second question*: What are VGIs? This has generated discussion over the past four decades. It is known that VGIs are biofilm-associated infections. Costerton et al. [16] confirmed the basic description of a biofilm which consists of single cells and microcolonies of sister cells embedded in a highly hydrated matrix including bacterial exopolymers and foreign macromolecules. The biofilm recruits bacteria resulting in further adherence [16].

VGEIs are then the result of a variety and diversity of factors of which intraoperative have a prominent role [14]. Biofilm has a fundamental role in the pathogenesis of VGEI and current research focus on how to prevent biofilm formation and how to chemically penetrate existing biofilms [17].

### Characteristics of VGIs

VGEIs share some characteristics.

1. They are common to all graft positions.
2. They have indolent pathogenic patterns with alternance of acute and quiescent periods.
3. They have an initial good response to antibiotic therapy; however, relapses are frequent because bacteria in biofilms are protected from antibiotics. As these bacterial foci go uncontrolled, the graft or device must be removed.
4. These infections are often polymicrobial, at least 30%. The predominant bacteria are autochthonous skin or bowel flora or common environmental microorganisms which are frequently pathogenic in immunocompromised patients [18].
5. Bacteria may not be easy to recover from fluids/tissues adjacent to grafts/devices.

### Evidence-based knowledge

Although there is substantial information accumulated in the literature, there still is no solid consensus on a number of aspects related to VGEI such as terminology, definition, classification, diagnostic criteria, and reporting standards. Complexity is a main issue in VGEI and it is frequently difficult to establish an accurate diagnosis and deciding which the best line of treatment is. In the case of intrathoracic VGEIs, the intrinsic risk of patients with VGI is usually the highest for the surgical patient. Due to the nature of the problem, there are no controlled studies and there is always mixed information coming from mixed territories. It is to be noticed that Clinical Practice Guidelines have not been issued until the last decade [11, 19, 20]. Part of the information is still being gathered from systematic reviews or Delphi Consensus documents [21, 22]. This once more highlights the difficulties of the clinicians when facing the problem of VGEI at any location. Furthermore, a large proportion of recommendations is of low level of evidence. Although useful information is being collected still at a low pace, the underlying message is that VGEI must include targeted antimicrobial therapy, radical debridement, and eventually prosthetic graft removal [10, 14]. The level of evidence is low as regards the best therapy and the replacement material.

## Prevention

As of today, and as stated above, current knowledge contemplates debridement, negative pressure wound therapy (NPWT) with continuous irrigation of the infected field. Reconstructive therapy whenever possible and systemic therapy were the options for management of VGEIs [10, 14]. It seems that there is a relationship between infection of the surgical site and prosthetic graft infection. Multiple approaches to prevent surgical site and graft infection have been tested in the laboratory setting and in clinical practice aiming at avoiding severe surgical trauma in already sick patients. Ikeno et al. proposed NPWT until system and regional negative cultures are achieved followed by reconstruction of the chest wall [23]. Conservative therapy has also been advocated by others [24, 25] in this setting. A major issue here is always reduction of bacterial load [26].

Having said that, prevention should start before something occurs, which may sound a bit naive. A major issue is if the prosthetic graft is itself a risk factor for postoperative infection. There are a number of issues to consider; first, the use of topical antibiotics as it has been advocated in the past may lead to bacterial resistance. This is a very old controversy active for over four decades [27]. Even today and although there is more literature available on surgical access through the groin, heterogeneity as regards access is a problem and there is paucity of data to support routine topical administration in surgical accesses [28].

Second, there is lack of robust long-term follow-up data on infection-free survival. Some mid-term outcomes in small-sized cohorts suggest that specific hybrid options in selected patients may yield safe outcomes for the treatment of infected vascular reconstructions [29]. More information is required over long periods of time to understand the eventual value of preventive measures of any kind on VGEI.

Third, studies focused on the use of antibiotic-bonded grafts to reduce the risk of VGEIs in vitro and in vivo. In large animal models, antimicrobial-bonded vascular graft material outperformed standard vascular grafts as regards graft patency and infection [30]. This animal study showed that their specific antibiotic composition with minocycline and rifampin with chlorhexidine precoating resisted to *Staphylococcus aureus* and limited the growth of *Pseudomonas aeruginosa*. However, clinical experience is heterogenous and diverse surveys show mixed opinions about preventive usage of antibiotic-bonded grafts [31, 32]. Furthermore, *S. aureus* has been shown to colonize rifampin-bonded grafts 7 days after implantation [33].

To further investigate the eventual role of the type of prosthesis and as part of previous work, an in vitro study was organized at the University Hospital Zurich, Switzerland, to compare the susceptibility of two thoracic vascular woven polyester grafts with different coatings to biofilm formation [34]. Implanted grafts are usually coated with proteinaceous solutions only, for quick integration into the host tissue. A collagen and a gelatin graft were compared. The collagen graft was coated with a highly purified form of cross-linked bovine type I collagen and the gelatin graft coated with a modified mammalian gelatin. The resorption time for collagen is 4–8 weeks and for gelatin, 14 days. In these in vitro experiments, grafts were dissected into small square pieces and were inoculated with bacterial strains representing pathogens implicated in thoracic VGEI from a patient cohort. Biofilm grown on collagen graft patches displayed increased total biofilm mass volume and maximal biofilm height, those finding supporting the potential of Gram-positive bacteria to adhere to collagen. Only minor affinity was observed for gelatin [34].

The in vivo study included 412 patients from the VASGRA cohort [10, 15]. Out of them, 28 developed intrathoracic VGI. The calculated percentage of intracavitary VGI was higher for patients in the collagen graft group [34]. Although there were some limitations intrinsic to the laboratory methodology, biofilm formation was increased on collagen-coated grafts in comparison with gelatin-coated. This may entail that graft material may be associated with a given VGI rate.

## Diagnosis

Over the past five decades, there has been abundant though heterogeneous literature on the topic of VGEI although as said earlier, recommendations and practice guidelines have been published in recent years due to the difficulties in organized controlled studies due to the complexity of the topic. The difficulties in diagnosis are multiple, from the protean manifestations of vascular infection to the various imaging methods available over time [35].

The diagnosis of VGEI is still a challenge for the practitioners as there are not clearly agreed standards. The diagnosis is established on some clinical and radiological criteria [36] and frequently the diagnosis is delayed which takes a toll on outcomes. Due to the lack of pre-established and validated criteria for VGI, some attempts have been made to extrapolate knowledge from other areas such as prosthetic valve endocarditis to VGEI [14, 37]. Very recently, a new case definition considering also major and minor criteria was proposed by the Management of Aortic Graft Infection Collaboration (MAGIC) [38]. The MAGIC criteria are meant to be a diagnostic standard for VGEI and have been well received by the community. Anagnostopoulos et al. [39] validated the MAGIC criteria by retrospectively evaluating the adjudicated VASGRA infection status [10]. Assuming the intrinsic limitations of a retrospective evaluation, it was found that the current MAGIC criteria offer good sensitivity and specificity in the context of infections; however, there was a slightly poorer sensitivity in VGEI than in other locations. The MAGIC criteria will in any case be a good tool for comparison of different management strategies [38].

**Fig. 1:**
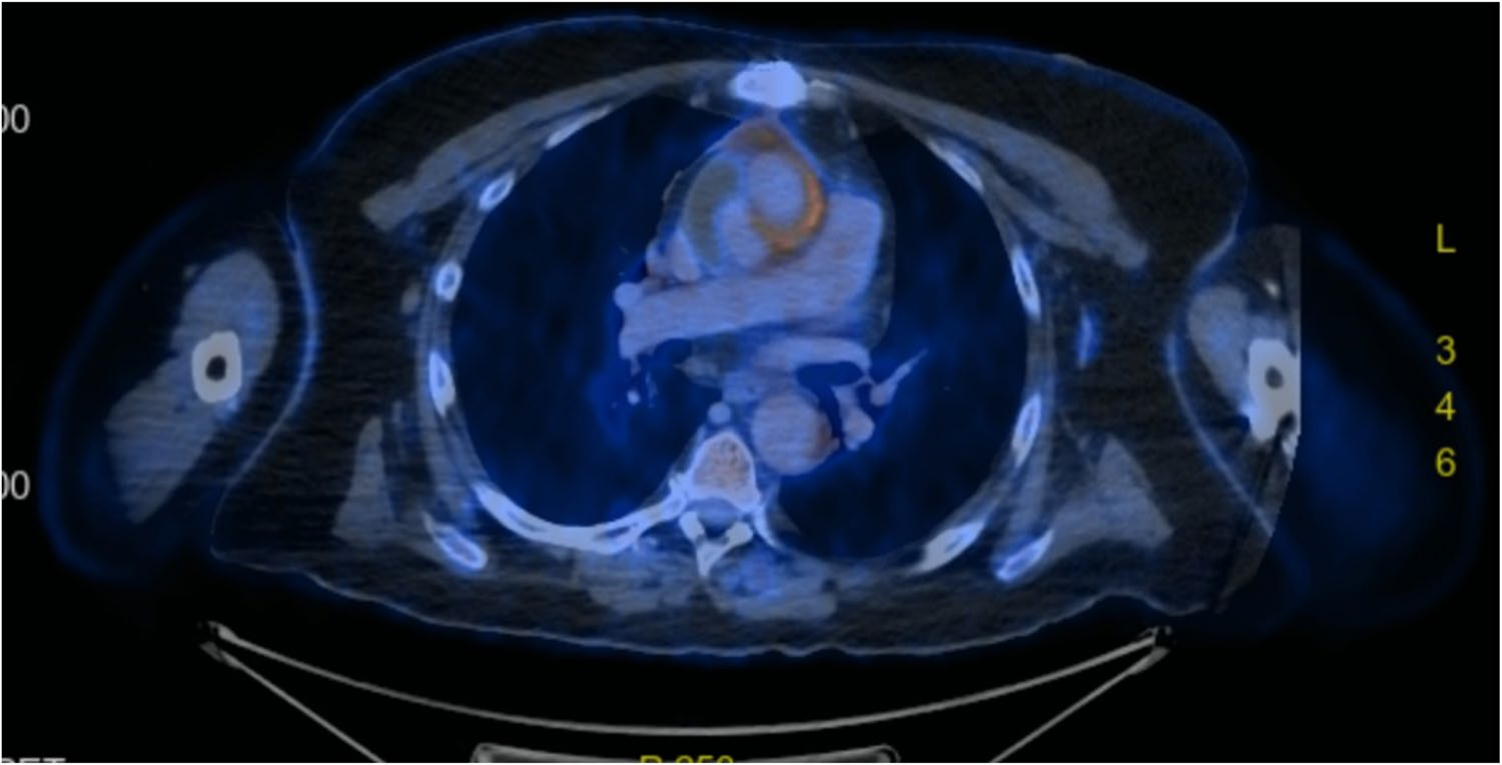
Preoperative positron emission tomography with 2-deoxy-2-[fluorine-18] fluoro-d-glucose (18F-FDG-PET/CT) showing uptake of an ascending aorta prosthetic graft highly suggestive of graft infection

Although the first-choice imaging modality is computed tomography [36], nuclear medicine methods such as positron emission tomography with 2-deoxy-2-[fluorine-18] fluoro-d-glucose, positron emission tomography, computed tomography (18F-FDG-PET/CT) are reliable non-invasive imaging modality for the diagnosis of primary vascular infection and VGEI [40] (Fig. 1). This has been confirmed over the years as PET/CT has added accuracy to the diagnosis of suspected vascular infection [41] and produced altering information in the therapy control of infective aneurysms [42] or established VGEIs [43]. Apparently, the diagnostic accuracy of 18F-FDG-PET/CT is higher than the accuracy of contrast-enhanced computed tomography (CE-CT) and demonstrates excellent sensitivity. The capacities of 18F-FDG-PET/CT beyond the actual body of a potentially infected graft are currently being explored and the role of abnormal locoregional lymph nodes in the diagnosis considered. According to van Rijsewijk et al., detection of abnormal nodes has a high specificity and positive predictive value for VGEI thus producing additional relevant information that might be incorporated as part of diagnostic criteria in the future [44].

## Antibiotic therapy

Antibiotic therapy is a major component of the management scheme in any major infection such as VGEI. Although guidelines provide strong recommendations in the case of infective endocarditis, a major bloodborne infection [45], unfortunately this is not the case in VGEI. One can then say that there are no universal recommendations on the usage of specific antibiotics for a given case of infection [14]. In our own experience [18], around one-third of VGI is polybacterial, meaning that both Gram-positive and Gram-negative pathogens should be tackled through a broad-spectrum combination of intravenous antibiotics until an accurate microbiological diagnosis is made; quite frequently this is not possible as all cultures may yield negative results. In the case of intrathoracic VGEI as we mostly refer here to, the combination of a beta-lactam (e.g., ampicillin-cloxacillin)/glycopeptide (e.g., vancomycin) and an aminoglycoside (e.g., gentamicin) [14]. The European and North American clinical practice guidelines produce slightly different recommendations according to the extent of resection and debridement as regards duration of antibiotic therapy [11, 19].

Regarding timing for surgery, the same problems as with which type and duration of therapy exist. The condition of the patient will dictate if a patient is eventually fit for surgery although clinical presentation will guide the treating team towards the most appropriate time to indicate a reoperative procedure of any kind. The case of, for instance, an aortoenteric fistula is clearly a surgical indication and the patient should be treated without major delay, unless specific contraindication is present.

## Replacement material

**Fig. 2:**
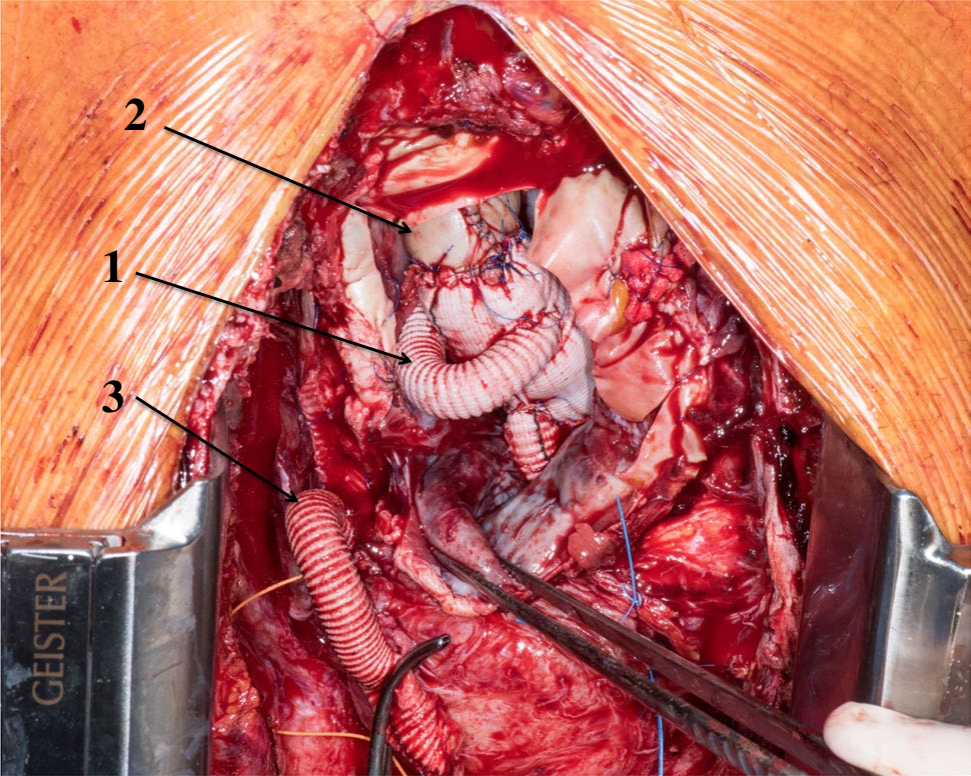
Intraoperative view after second reoperation for infection of an ascending aorta prosthetic graft. In a complex procedure, the patient underwent aortic root re-replacement with a prosthetic valved conduit requiring coronary management with separate 10-mm prosthetic grafts to the left and the right coronary arteries (1). Distal to the prosthetic graft, a self-made xenopericardial tube was used to reach the aortic arch (2). The patient had a previous Cabrol fistula to the right atrium (fine arrow) from an explanted mediastinal patch (3)

There have been different opinion streams as regards the value and role of a diversity of graft materials in vascular surgery and especially in the field of VGEIs. Currently, commercially available prosthetic grafts and vascular devices are mainly made of two different materials such as polymers, polyethylene terephthalate (PET) (Fig. 2) and expanded polytetrafluoroethylene (ePTFE), and different alloys, nitinol being the most used worldwide [19]. Little has been investigated as regards potential infectability of these materials, which are chosen because of their chemical and mechanical properties [17, 34, 46]. Comparative studies in graft infections yielded conflictive results as different models have been used. Rowe et al. created a porcine model of thoracic aortic graft infection [47] to compare the ability of cryopreserved arterial allografts (CAA) to resist infection in comparison with collagen-impregnated Dacron grafts (CIDG). Animals received *S. aureus* boluses and were euthanized after 8 weeks. Interestingly, in this animal model, CIDG has an infection rate of 16.7% and cryopreserved allografts 57.2%, although there were no statistically significant differences. On the other hand, Vogt et al. in a series of 72 patients suffering from different types of vascular infection compared the outcomes of CAA with conventional vascular prostheses [48]. The use of CAA was more effective in the treatment of mycotic aneurysms and infected vascular prostheses in terms of disease-related survival, disease-related survival free of reoperation, hospitalization, duration of antibiotic therapy, and elimination of infection at 5 years postoperatively. The use of CAA offers a promising solution in thoracic and thoracoabdominal infection due to their apparently superior resistance to infection although they are not free from late complications [18, 49–51]. However, it is still unclear if there is a superior replacement option on the long-term [52].

A newer surgical concept was introduced and discussed for the treatment of thoracic and abdominal graft infections. Czerny et al. evaluated the use of self-made xenopericardial tube grafts constructed from a patch [52] in an initial series of 15 patients with VGEI. Two years postoperatively, freedom from reinfection and reoperation was 100%. The authors concluded that these xenopericardial tube grafts as neoaortic segments produced good results and represented an alternative to CAA. Additional information was released in 2018 by Kreibich et al. [53] and confirmed the same initial results. Perioperative mortality in all these series is still high considering the frail state of patients with VGEI; the concept has been validated across different series with focus on the thoracic and thoracoabdominal aorta with proven promising data in the short-term [54–56] regarding freedom from reinfection and graft durability. Additional data with focus on follow-up structural issues are mandatory.

## Adjunctive procedures in the case of VGI

The role of adjunctive procedures is still unclear and controversial as of today. Some of them have been advocated in the literature for long time; however, no consensus has been reached with this regard. The greater omentum is a highly vascularized tissue, being directly supplied from the right and left gastroepiploic arteries and has been used in a number of different procedures in the abdomen and in the chest. The greater omentum has been reported as a major and useful barrier against infection in abdominal and thoracic aneurysm surgery. In line with this assumption, wrapping of grafts has been advocated to prevent infection [57]. In any case, the greater omentum is a versatile biological material that can be used in complex thoracic and abdominal conditions including VGI [58].

Muscle flaps have been used in a variety of reconstructive procedures and also to treat VGI being this an old story [59]. Muscle flaps have been transferred mainly to the inguinal regions to cover wounds and/or vascular grafts with excellent results in terms of graft salvage [60, 61].

## Limitations

This is just a narrative review of the literature with no systematic search. This may have influence on opinions.

## Conclusions

VGI is a serious condition that entails high morbidity and mortality. Despite advancements in the field, there is still lack of consensus as regards the type of replacement material. Prevention of VGEI is complex as several pre- and intraoperative factors play a role in the pathogenesis of prosthetic infection. Advances in diagnosis that include nuclear medicine techniques seem to help in the diagnosis of VGEI and have gained momentum in the past decade. There is a need to collect larger series with prolonged follow-up.
